# Urine-sample-derived human induced pluripotent stem cells as a model to study PCSK9-mediated autosomal dominant hypercholesterolemia

**DOI:** 10.1242/dmm.022277

**Published:** 2016-01-01

**Authors:** Karim Si-Tayeb, Salam Idriss, Benoite Champon, Amandine Caillaud, Matthieu Pichelin, Lucie Arnaud, Patricia Lemarchand, Cédric Le May, Kazem Zibara, Bertrand Cariou

**Affiliations:** 1 INSERM, UMR1087, L’institut du thorax, Nantes F-44000, France; 2 CNRS, UMR 6291, Nantes F-44000, France; 3 Université de Nantes, Nantes F-44000, France; 4 CHU Nantes, L’institut du thorax, CIC Endocrinology-Nutrition, Nantes F-44000, France; 5 ER045 - Laboratory of Stem Cells, PRASE, DSST, Beirut, Lebanon; 6 Biology Department, Faculty of Sciences-I, Lebanese University, Beirut 6573/14, Lebanon

**Keywords:** Urine-derived somatic cells, Human induced pluripotent stem cells, Hepatocyte differentiation, Autosomal dominant hypercholesterolemia, PCSK9

## Abstract

Proprotein convertase subtilisin kexin type 9 (PCSK9) is a critical modulator of cholesterol homeostasis. Whereas *PCSK9* gain-of-function (GOF) mutations are associated with autosomal dominant hypercholesterolemia (ADH) and premature atherosclerosis, *PCSK9* loss-of-function (LOF) mutations have a cardio-protective effect and in some cases can lead to familial hypobetalipoproteinemia (FHBL). However, limitations of the currently available cellular models preclude deciphering the consequences of PCSK9 mutation further. We aimed to validate urine-sample-derived human induced pluripotent stem cells (UhiPSCs) as an appropriate tool to model PCSK9-mediated ADH and FHBL. To achieve our goal, urine-sample-derived somatic cells were reprogrammed into hiPSCs by using episomal vectors. UhiPSC were efficiently differentiated into hepatocyte-like cells (HLCs). Compared to control cells, cells originally derived from an individual with ADH (HLC-S127R) secreted less PCSK9 in the media (−38.5%; *P*=0.038) and had a 71% decrease (*P*<0.001) of low-density lipoprotein (LDL) uptake, whereas cells originally derived from an individual with FHBL (HLC-R104C/V114A) displayed a strong decrease in PCSK9 secretion (−89.7%; *P*<0.001) and had a 106% increase (*P*=0.0104) of LDL uptake. Pravastatin treatment significantly enhanced LDL receptor (*LDLR*) and *PCSK9* mRNA gene expression, as well as PCSK9 secretion and LDL uptake in both control and S127R HLCs. Pravastatin treatment of multiple clones led to an average increase of LDL uptake of 2.19±0.77-fold in HLC-S127R compared to 1.38±0.49 fold in control HLCs (*P*<0.01), in line with the good response to statin treatment of individuals carrying the S127R mutation (mean LDL cholesterol reduction=60.4%, *n*=5). In conclusion, urine samples provide an attractive and convenient source of somatic cells for reprogramming and hepatocyte differentiation, but also a powerful tool to further decipher PCSK9 mutations and function.

## Abstract (precis)

**Summary:** The authors used urine-sample-derived patient-specific human induced pluripotent stem cells to generate hepatocytes carrying gain- or loss-of-function mutations of PCSK9, and mimicking the pathophysiology *in vitro*.

## INTRODUCTION

Proprotein convertase subtilisin kexin type 9 (PCSK9) has been identified as a major regulator of cholesterol homeostasis (Costet et al., 2008; Seidah et al., 2003; Seidah et al., 2014). PCSK9 is subjected to an intracellular autocleavage before being secreted by the liver into the plasma, where it acts as an endogenous inhibitor of the low-density lipoprotein (LDL) receptor (LDLR). PCSK9 binds the extracellular epidermal growth factor-like repeat A (EGF-A) domain of the LDLR and thus disrupts its recycling to the cell surface by targeting it to the lysosomal pathway for degradation (Seidah et al., 2014). In their seminal paper, Abifadel and colleagues demonstrated that a *PCSK9* gain-of-function (GOF) mutation is the third most common cause of autosomal dominant hypercholesterolemia (ADH), a genetic disease associated with premature atherosclerosis (Abifadel et al., 2003). Thereafter, whole-life lower concentrations of plasma LDL-cholesterol (LDL-C) and protection against cardiovascular diseases were associated with *PCSK9* loss-of-function (LOF) mutations (Cohen et al., 2006). In some cases, *PCSK9* LOF mutations can lead to familial hypobetalipoproteinemia (FHBL) (Cariou et al., 2009; Zhou et al., 2012). Thus, lowering levels of LDL-C through PCSK9 inhibition together with statins has been considered as an attractive and promising pharmaceutical approach (Cariou et al., 2011; Farnier, 2013). In order to reach this goal, human monoclonal antibodies (mAbs) directed against PCSK9 were developed for clinical applications and recent Phase 3 trials showed a strong reduction (up to 60%) of plasma LDL-C concentrations in various hypercholesterolemic patients treated with anti-PCSK9 mAb (Blom et al., 2014; Cannon et al., 2015; Cariou et al., 2011; Farnier, 2013; Roth and Diller, 2014).

To date, current cellular models that are available to decipher PCSK9 functions are limited to heterologous cell lines or hepatoma cells with forced PCSK9 expression that are not suitable to clearly describe the intracellular action of PCSK9. Alternatively, the physiological relevance of cultured human fibroblasts for studying lipoprotein metabolism has recently been challenged (Sniderman et al., 2013). Therefore, we propose that the use of human induced pluripotent stem cells (hiPSCs) differentiated into hepatocyte-like cells (HLCs) could provide an adapted cellular environment to conduct such studies. Indeed, it has been shown that hiPSCs generated from skin fibroblasts of an individual carrying mutations in the *LDLR* gene and differentiated into HLCs were able to recapitulate the features of ADH (Cayo et al., 2012; Si-Tayeb et al., 2010). Here, we used a convenient source of patient-derived somatic cells isolated directly from their urine samples. Urine-derived progenitor cell (Ucell) isolation and amplification (for a review, see Gerbal-Chaloin et al., 2014), as well as their reprogramming into hiPSCs (Xue et al., 2013; Zhou et al., 2012), has been previously established. Our study reports for the first time the modeling of PCSK9-mediated ADH and FHBL after hepatic differentiation of hiPSCs reprogrammed from urine-derived somatic cells, therefore establishing a new patient-related model to further decipher PCSK9 functions.

## RESULTS

### Urine sample as a source of human somatic cells

**Fig. 1.**
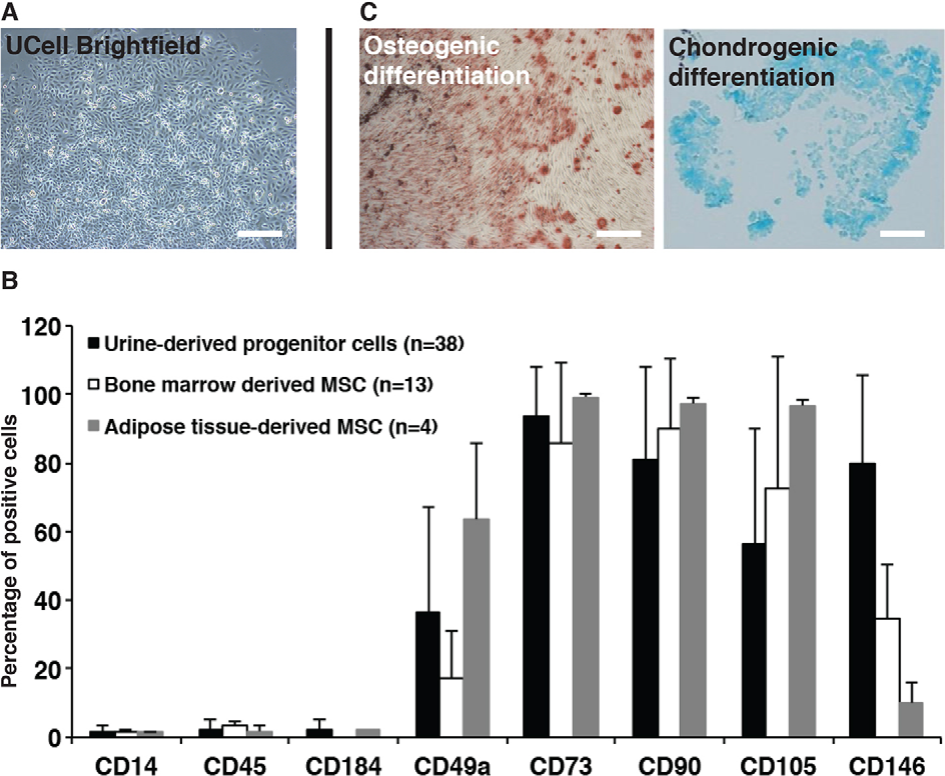
**Ucell characterization.** (A) Urine-derived progenitor cell (Ucell) morphology after isolation and amplification. (B) Cell marker expression analysis (percentage of positive cells) of Ucells, bone-marrow-derived and adipose-tissue-derived mesenchymal stem cells by flow cytometry. (C) Osteoblastic and chondrogenic differentiation of Ucells detected by Alizarin Red and Alcian Blue staining, respectively. Scale bars: 100 µm.

Ucells were isolated and amplified from urine samples of 38 donors (healthy or with ADH or FHBL). After a week of culture, clusters of Ucells displayed a ‘rice grain’ morphology with a high rate of proliferation (Fig. 1A) in 67% of collected healthy donors or patients, including those carrying the PCSK9-S127R (ADH) and R104C/V114A (FHBL) mutations. As observed with bone-marrow- and adipose-tissue-derived mesenchymal stromal cells (MSCs) by flow cytometry, Ucells did not express the hematopoietic markers CD14, CD45 and CD184, and expressed MSC markers CD49a, CD73, CD90 and CD105 (Fig. 1B). We also noticed a higher number of Ucells positive for the endothelial-cell-lineage marker CD146 compared to MSCs. Finally, the ability of Ucells to perform osteoblastic and chondrogenic differentiation was verified (Fig. 1C). Control Ucells from a healthy female donor, PCSK9-S127R Ucells from a female patient, initially described in the seminal paper identifying PCSK9-mediated ADH (Abifadel et al., 2003), and Ucells PCSK9-R104C/V114A from a male patient with FHBL (Cariou et al., 2009) were used for subsequent experiments.

### Generation and characterization of UhiPSCs

Ucells were reprogrammed upon transfection of factors encoded by episomal vectors. Several stoichiometries were tested (Fig. S1) to select the most efficient cocktail of reprogramming factors to generate urine-sample-derived human induced pluripotent stem cells (UhiPSCs). Several clones were obtained from control, S127R and R104C/V114A Ucells, and three of each were used for hepatocyte differentiation and characterization.

**Fig. 2.**
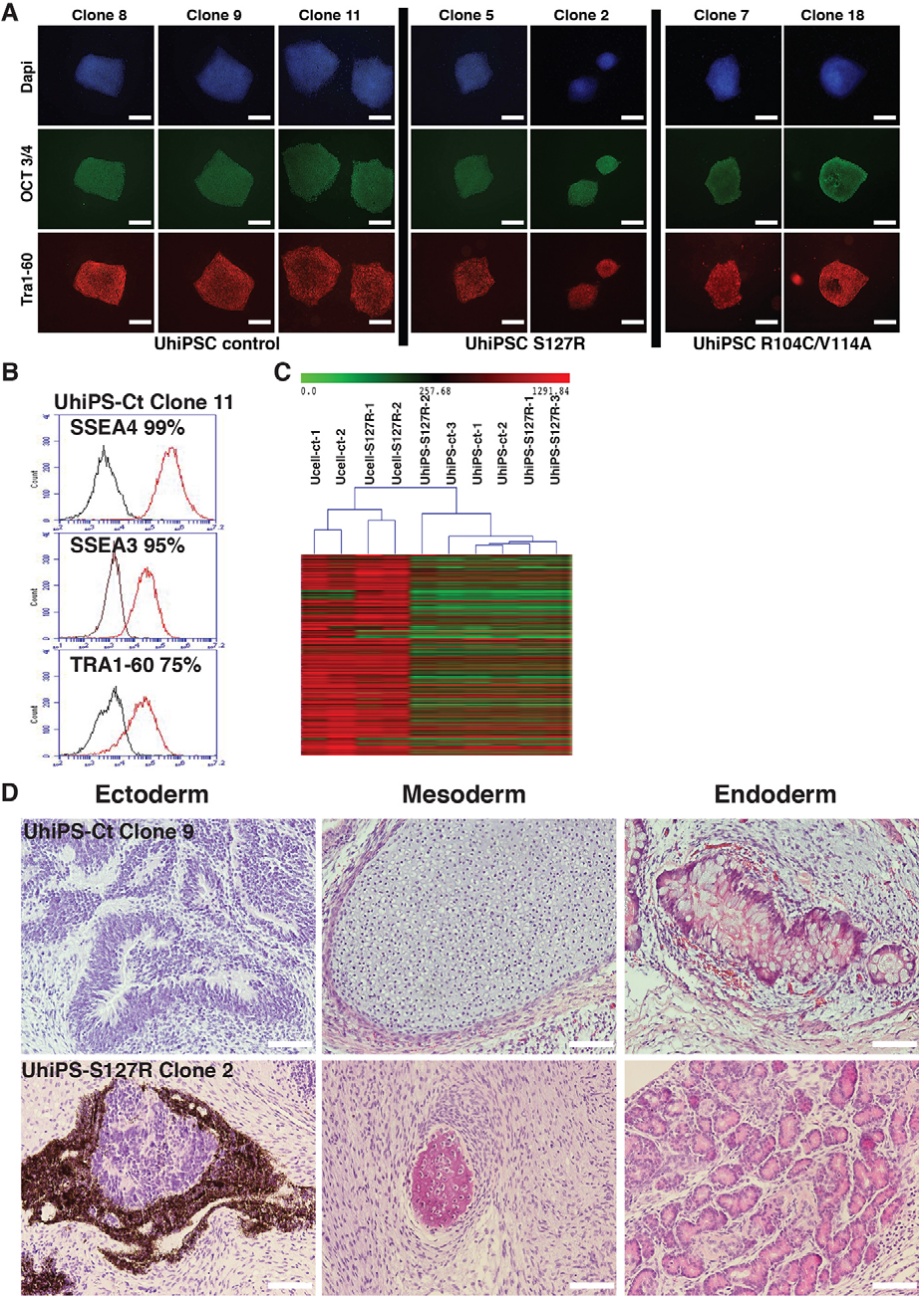
**UhiPSC** **characterization.** (A) Detection of OCT3/4 and Tra1-60 expression by fluorescent immunostaining on control UhiPSCs and UhiPSCs carrying the PCSK9-S127R or PCSK9-R104C/V114A mutations. (B) Representative flow-cytometry analysis for the detection of SSEA4, SSEA3 and TRA1-60 expression by control (Ct) UhiPSCs. (C) Global gene-expression profile comparison between Ucell control (ct), Ucell S127R, UhiPSC control and UhiPSCs carrying the PCSK9-S127R mutation. (D) Hematoxylin- and eosin-stained histological section of teratomas showing, from left to right, ectoderm-derived neurons, mesoderm-derived cartilage and endoderm-derived intestinal-like tissue for UhiPSC-Control (Ct), and ectoderm-derived retinal cells, mesodermal-derived bone tissue and endodermal-derived exocrine pancreatic glands for UhiPSC PCSK9-S127R. Scale bars: 100 µm.

Control, PCSK9-S127R and PCSK9-R104C/V114A UhiPSCs formed colonies with a morphology similar to that of human embryonic stem cells on mouse embryonic fibroblasts (MEFs). They expressed the pluripotency markers OCT3/4 and TRA1-60, as assessed by immunofluorescence (Fig. 2A), as well as SOX2 and NANOG, as assessed by quantitative reverse-transcriptase PCR (RT-qPCR); the loss of episomal vectors was also verified (Fig. S2A). Ucells and corresponding UhiPSCs shared the same DNA fingerprint (data not shown), and, compared to control cells, PCSK9-S127R and PCSK9 R104C/V114A UhiPSCs retained their PCSK9 mutation upon reprogramming (Fig. S2B). Under feeder-free conditions, tested clones (control, PCSK9-S127R and PCSK9-R104C/V114A) remained positive for the pluripotency markers SSEA4, SSEA3 and TRA1-60 following flow cytometry analysis (Fig. 2B and Fig. S2C). A transcriptomic analysis was performed and confirmed the global gene-expression profile modification between two control Ucell lines, two PCSK9-S127R Ucell lines and three clones of each UhiPSC line generated (Fig. 2C). The injection of control and PCSK9-S127R UhiPSCs into NOD Scid gamma (NSG) mice led to the formation of teratomas that included differentiated tissues from all three germ layers (Fig. 2D). Finally, although reprogrammed cells displayed normal karyotypes (Fig. S3), trisomy (chromosomes 1, 14 or 21) occurred in some clones at late passages (above p65; data not shown). Therefore, UhiPSCs between passage 25 and 40 were used for further differentiation and functional analyses. Altogether, our data support the successful reprogramming of Ucells.

### UhiPSCs differentiate into hepatocyte-like cells

**Fig. 3.**
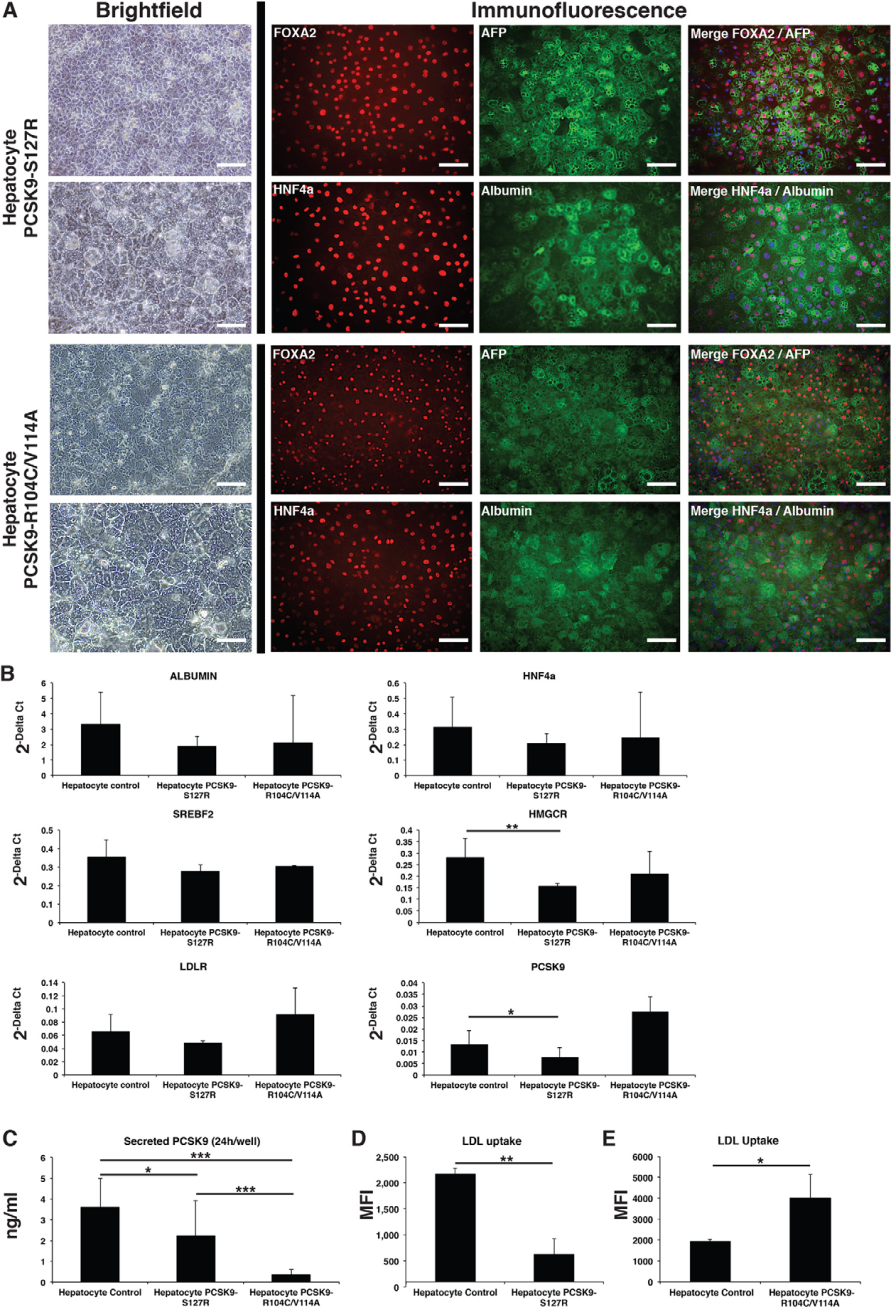
**UhiPSC** **differentiation into** **HLCs.** (A) Pictures of HLCs carrying the S127R or R104C/V114A mutations. Left: HLC morphology observed by brightfield microscopy (the lower panel represents a magnification of the upper panel). Right: detection of the hepatic markers FOXA2, AFP, HNF4α and albumin by fluorescent immunostaining (nuclei were stained in blue in merged pictures). Scale bars: 100 µm. (B) Gene expression analysis by RT-qPCR of albumin, *HNF4a*, *SREBF2*, *LDLR*, *PCSK9* and *HMGCR* in control, S127R and R104C/V114A HLCs (one clone per UhiPSC line; control *n*=6 differentiations, PCSK9-S127R *n*=3 differentiations, PCSK9-R104C/V114A *n*=3 differentiations). (C) Secreted PCSK9 detection by ELISA assay (one clone per UhiPSC line; control *n*=9 differentiations, PCSK9-S127R *n*=9 differentiations, PCSK9-R104C/V114A *n*=14 differentiations). (D,E) Quantification by flow cytometry of incorporated LDL from HLCs (D) PCSK9-S127R and (E) R104C/V114A compared to controls. MFI, mean fluorescence intensity. *P<0.05, **P<0.01, ***P<0.001.

UhiPSCs were differentiated toward HLCs as a monolayer under feeder-free conditions and completely defined medium. Control (not shown), PCSK9-S127R and PCSK9-R104C/V114A differentiated cells displayed human hepatocyte morphology and co-expressed the hepatic markers FOXA2, AFP, HNF4α and albumin, as seen by immunofluorescent staining (Fig. 3A). By counting the albumin-positive cells, the differentiation efficiency was evaluated to 84.9±7%, 92.7±1.2% and 91.4±4.8% for control, PCSK9-S127R and PCSK9-R104C/V114A HLCs, respectively. Gene expression levels of several hepatic genes, such as *ALB*, *HNF4a* and *SREBF2*, as well as *LDLR*, were similar in control, S127R and R104C/V114A HLCs. A slight, but significant, reduction of *PCSK9* mRNA level (41.9±31%; *P*=0.046) and a reduction of *HMGCR* mRNA levels (46±4.2%; *P*=0.003) were observed in S127R HLCs compared to control HLCs (Fig. 3B).

### Differentiated UhiPSCs recapitulate the functional defects of PCSK9-S127R and PCSK9-R104C/V114A mutations

Next, we aimed to demonstrate that UhiPSCs could serve as an appropriate cellular model to study PCSK9 mutations. In accordance with the inability of the mutated PCSK9 protein encoded by the S127R allele to be secreted extracellularly (Benjannet et al., 2004; Cameron et al., 2006), we found a 38.5% reduction of PCSK9 secretion by PCSK9-S127R HLCs compared to controls (Fig. 3C). In addition, a drastic 89.7% decreased level of PCSK9 secretion was observed in R104C/V114A HLCs compared to controls (Fig. 3C). This is in line with the dominant negative effect exerted by R104C/V114A previously reported *in vitro* and the absence of detectable circulating PCSK9 in the corresponding affected individual (Cariou et al., 2009). In accordance with the hypercholesterolemic phenotype of S127R carriers, HLC PCSK9-S127R showed a 71% (±14%; *P*<0.001) decrease in their ability to uptake LDL compared to HLC controls, as assessed by fluorescent microscopy (Fig. S4) and by flow cytometry analysis (Fig. 3D). In contrast, as expected with a LOF PCSK9 mutation, HLC PCSK9-R104C/V114A displayed a 106% (±57%; *P*=0.0104) increase in their ability to uptake LDL as compared to HLC control (Fig. 3E). Our results demonstrated that HLCs differentiated from hiPSCs derived from urine samples of individuals with PCSK9-mediated ADH or FHBL reproduced the main PCSK9 pathological features *in vitro*.

### PCSK9-S127R HLCs displayed an increased response to statin

**Fig. 4.**
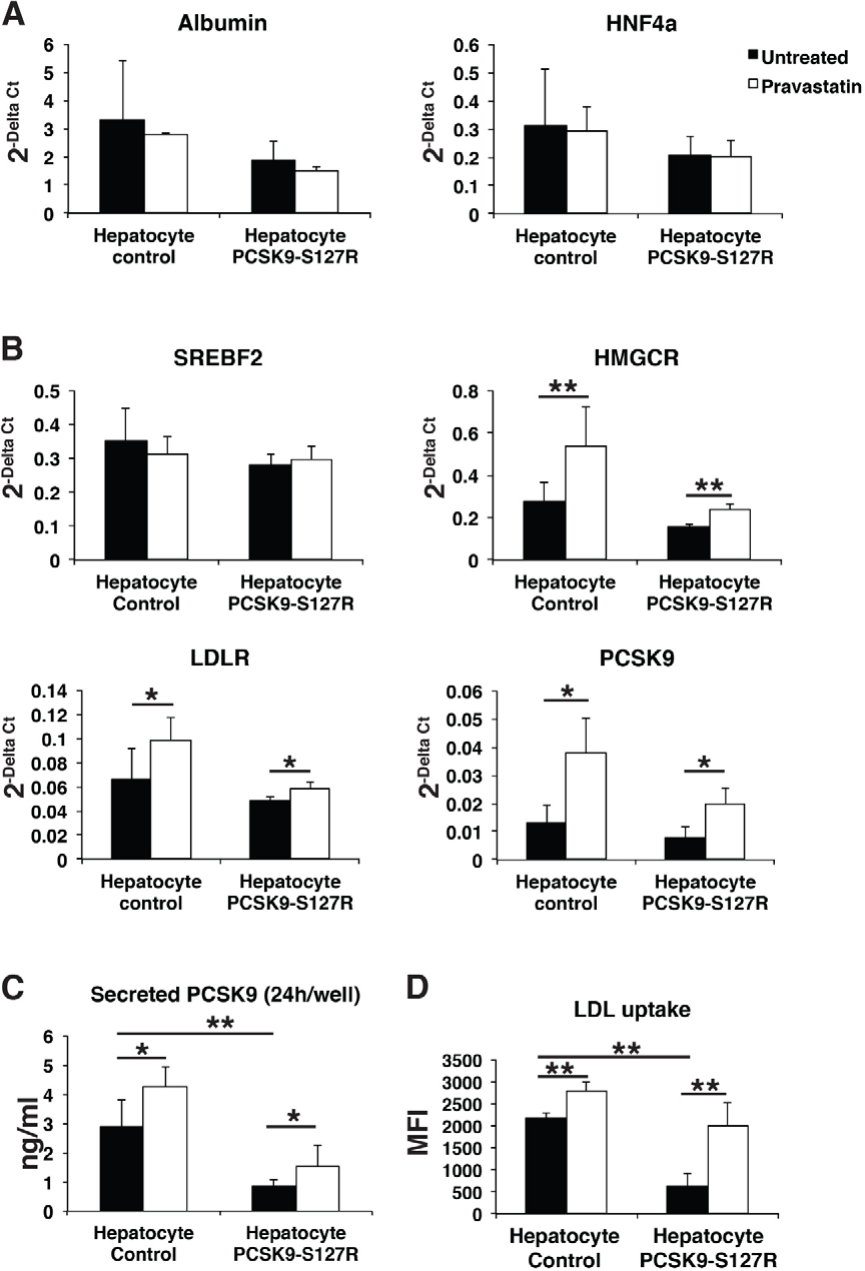
**Differentiated cell response to pravastatin treatment.** (A,B) Gene expression analysis by RT-qPCR of albumin, *HNF4a*, *SREBF2*, *LDLR*, *PCSK9* and *HMGCR* in an untreated condition (black bars) and after 24 h of pravastatin treatment at 10 µM (white bars) of control and S127R HLCs (one clone per UhiPSC line; control *n*=6 differentiation, PCSK9-S127R *n*=3 differentiation). (C) Secreted PCSK9 detection by ELISA assay (one clone per UhiPSC line; control *n*=6 differentiations, PCSK9-S127R *n*=6 differentiations). (D) Detection of incorporated LDL by flow cytometry (one clone per UhiPSC line; control *n*=6 differentiations, PCSK9-S127R *n*=3 differentiations). MFI, mean fluorescence intensity. *P<0.05, **P<0.01.

**Fig. 5.**
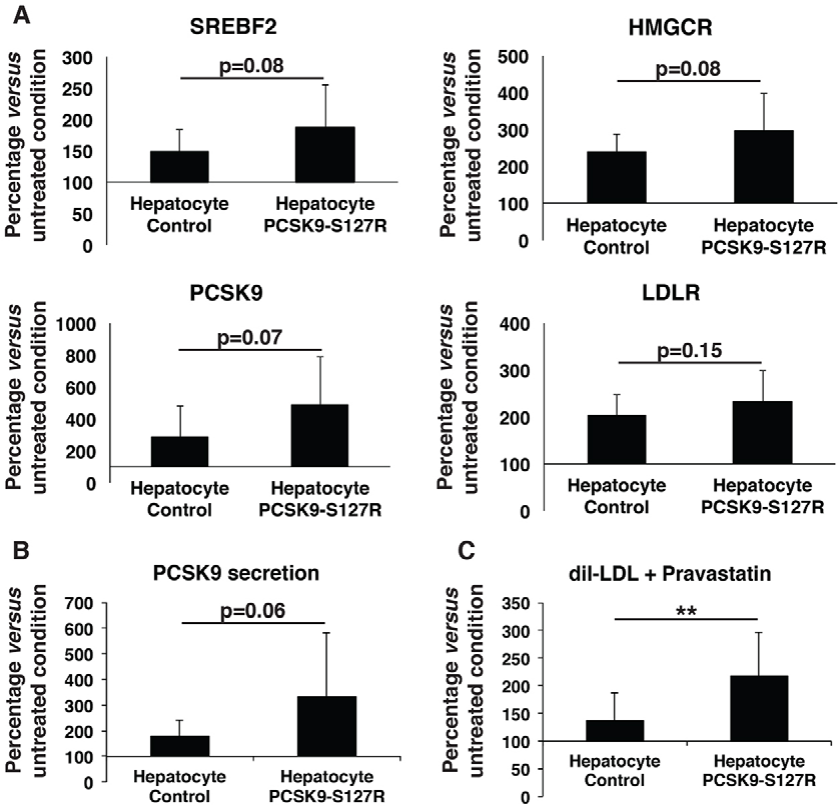
**Comparative analysis of the response of control and PCSK9-S127R HLCs to pravastatin treatment, normalized to untreated conditions.** (A) Gene expression analysis by RT-qPCR of *SREBF2*, *LDLR*, *PCSK9* and *HMGCR* (three clones per UhiPSC line; *n*=3 differentiations per clone). (B) Secreted PCSK9 detected by ELISA assay (one clone per UhiPSC line; *n*=9 differentiations per clone). (C) Detection of incorporated LDL by flow cytometry (three clones per UhiPSC line; *n*=3 differentiations per clone). **P<0.01.

In a final step, we investigated the effect of statins, the most widely used hypolipidemic drugs in clinical practice. Statins act by inhibiting 3-hydroxy-3-methyl glutaryl coenzyme A (CoA) reductase (HMGCR), leading to reduced endogenous cholesterol synthesis and subsequent upregulation of LDLRs through the SREBP2 pathway. Control and PCSK9-S127R HLCs were treated with pravastatin (10 µM) for 24 h, which did not affect the gene expression of hepatic markers such as *HNF4a* and albumin (Fig. 4A). Whereas *SREBF2* expression was not modified by the pravastatin treatment, because its regulation is mostly post-transcriptional, a significant increase in SREBP2 target gene expressions was observed, including *HMGCR* (control: 93±66%; PCSK9-S127R: 53±16%), *LDLR* (control: 50±28%; PCSK9-S127R: 21±11%) and *PCSK9* (control: 185±95%; PCSK9-S127R: 157±74%) (Fig. 4B). Correlated with these gene expression data, an increase in PCSK9 secretion was observed by 48.8±23% and 86.3±87% (Fig. 4C) and LDL uptake by 25.2±8% and 352.9±137% (Fig. 4D) in control and S127R HLCs, respectively. Similar results were observed upon lovastatin (10 µM) treatment (data not shown). These results indicate that UhiPSC-derived HLCs displayed all the characteristics necessary to study PCSK9 functions in a suitable cellular environment. To further validate these results, the same experiments were repeated in two different additional clones of control and PCSK9-S127R UhiPSCs. Compared to controls, HLC PCSK9-S127R displayed non-significant enhanced expression of *SREPF2*, *HMGCR*, *PCSK9* and *LDLR* mRNA levels under pravastatin treatment (Fig. 5A). In addition, the induction of PCSK9 secretion upon statin treatment was more pronounced in HLC S127R than in controls (Fig. 5B). Finally, the statin-induced LDL uptake in HLCs carrying the PCSK9-S127R mutation was significantly higher than in control HLCs [2.19±0.77-fold (*n*=20) vs 1.38±0.49-fold (*n*=26), respectively; *P*<0.01] (Fig. 5C).

Although unexpected, our results indicated that the PCSK9-S127R mutation was associated with an enhanced response to statins *in vitro* in HLCs.

### Individuals carrying the S127R mutation are good responders to statins

In order to validate the clinical relevance of these *in vitro* results obtained in HLCs, we investigated the initial response to statin therapy in five female patients with ADH related to S127R mutations. As shown in Table 1, the mean decrease of LDL-C following statin treatment was 60.4% [confidence interval (CI): −56 to −64%]. Amongst these patients, 3/5 were under a high dose of statins (i.e. rosuvastatin 20 mg once daily). These data clearly indicate that individuals with S127R mutations are good responders to statin therapy, strengthening the usefulness of UhiPSC-derived HLCs to model PCSK9-mediated ADH.

**Table 1.**
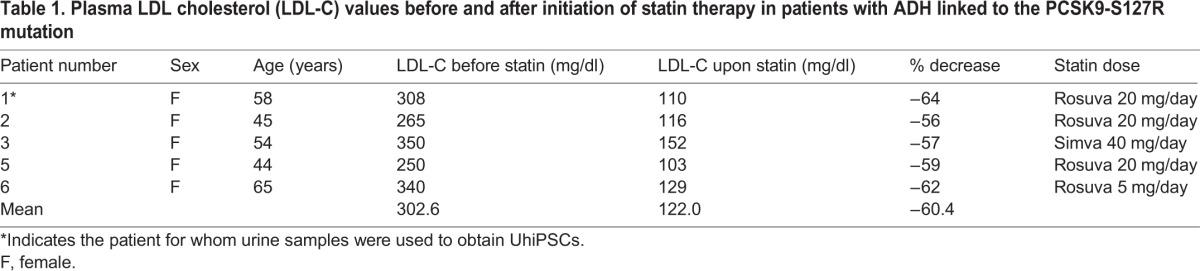
**Plasma LDL cholesterol (LDL-C) values before and after initiation of statin therapy in patients with ADH linked to the PCSK9-S127R mutation**

## DISCUSSION

This study not only highlights the usefulness of urine samples as a convenient source of patient-derived somatic cells to generate UhiPSCs, but also demonstrates their potential, when differentiated into HLCs, to model human metabolic diseases, such as ADH. Notably, we demonstrated for the first time that HLCs are a unique tool to study PCSK9 biology. Here, we specifically studied the seminal PCSK9-S127R GOF mutation that allowed the identification of PCSK9 as the third gene of ADH in a large French family in 2003 (Abifadel et al., 2003).

The S127R mutation is one of the most intriguing PCSK9 ones, which, despite its inability to be secreted in the extracellular space, provokes ADH, suggesting an intracellular role of PCSK9 (Benjannet et al., 2004; Pandit et al., 2008). Compared to the D374Y GOF mutation, which strengthens the affinity between PCSK9 and LDLR, the S127R mutation does not affect PCSK9 binding to LDLR (Pandit et al., 2008). It has been suggested that the S127R mutation might either enhance the ability of PCSK9 to bind LDLR after cellular uptake or might act intracellularly to redirect LDLR to the lysosomes for degradation. However, it is important to note that discrepant data have been reported concerning the impact of S127R mutation on the amount of cell surface LDLR and/or internalization rate of LDL by LDLR. On one hand, reduced amounts of LDLR and decreased internalization of LDL have been described in HepG2 cells stably transfected with S127R when compared with wild-type (WT)-PCSK9 (Benjannet et al., 2004). On the other hand, the overexpression of S127R and WT-PCSK9 in HepG2 cells or in mouse livers using adenoviruses did not lead to statistical differences in regards to the amount of LDLR (Park et al., 2004). In addition, the McArdle RH7777 hepatoma cell line stably expressing PCSK9-S127R constructs failed to show a significant reduction of the amount of LDLR when compared to those expressing WT-PCSK9 (Sun et al., 2005).

Our data suggested that less PCSK9 was secreted from S127R compared to control HLCs, in accordance with a defect in PCSK9 maturation and processing. However, this observation has been made from multiple differentiations of a single UhiPSC clone generated from one individual carrying the PCSK9-S127R GOF mutation, and suffered from a lack of statistical robustness (*P*=0.038). Therefore, PCSK9 secretion must be evaluated from HLCs obtained from multiple donors carrying the PCSK9-S127R mutation in order to strengthen and confirm our data. Importantly, the ability of HLC S127R to internalize LDL is severely reduced by 71% compared to control cells, validating the hypothesis of an additional intracellular pathway for the regulation of LDLR trafficking by PCSK9. Our results obtained with UhiPSC-derived HLCs confirmed those observed in lymphoblasts of individuals with the S127R mutation, in which the cell surface LDLR levels are reduced by 35% (Benjannet et al., 2004). In contrast, in HepG2 cells transfected with PCSK9-S127R plasmid, only a 7% reduction in the amount of LDL internalized is observed when compared with WT-PCSK9 (Cameron et al., 2006). It could be reasonably hypothesized that endogenous partners and/or regulators of PCSK9 are expressed in a more physiological stoichiometry in HLCs than in lymphoblasts or transfected hepatoma cell lines. Furthermore, although the use of lymphoblasts seems attractive because of their ease to be generated compared to differentiated UhiPSCs, it has been reported that the expression level of PCSK9 in lymphocytes is less than 1/1000 of the level reported in hepatocytes (Holla et al., 2006). The liver remains the major organ involved in PCSK9 expression and secretion (Zaid et al., 2008) and the regulation of the cholesterol metabolism. Thus, investigating PCSK9 roles and functions in lipid disorders such as ADH and FHBL would be more likely to be reliable if it is done in the context of human-derived hepatocytes (i.e. HLCs).

Because control and mutated HLCs are coming from unrelated donors, the different genetic background carried by the different UhiPSCs could hamper our observations. Therefore, to further validate our model, we generated UhiPSCs from an individual carrying heterozygous LOF mutations R104C/V114A. Upon HLC differentiation, and in line with previously published data (Cariou et al., 2009), we observed a strong decrease in PCSK9 secretion (89.7%) together with an increased LDL uptake (106%). Altogether, our data strongly suggest that the phenotypes observed with the use of our model are linked to PCSK9 mutations rather than genetic-background differences.

UhiPSC-derived HLCs could serve as a valuable pharmacological cell model because they respond normally to the hypolipidemic drugs statins. As expected, statin treatment induces: (i) expression of SREBP2 target genes such as *LDLR* and *PCSK9*; (ii) PCSK9 secretion; and (iii) LDL uptake. Unexpectedly, HLCs carrying the S127R GOF mutation displayed a better response to statin, with a more important increase of LDL uptake compared to control cells. This finding was counterintuitive because both PCSK9-deficient mice (Rashid et al., 2005) and individuals harboring *PCSK9* LOF mutations (Berge et al., 2006) have an increased response to statin therapy. Importantly, our *in vitro* observations correlated with clinical observations from individuals carrying the S127R mutation, who seem to be good responders to statins, with a mean decrease of LDL-C by ≈60%. Usually, a reduction in serum LDL-C of 52% is observed on rosuvastatin 20 mg and 38-40% on simvastatin 40 mg in non-ADH patients (Jones et al., 1998, 2003). The underlying molecular mechanisms of this increased sensitivity to statins related to S127R mutation remain unknown, and need further investigations. Although there was a tendency for an increase of *SREBF2* expression as well as that of its main target genes, *HMGCR*, *PCSK9* and, to a lesser extent, *LDLR*, in S127R HLCs compared with controls, it did not reach statistical significance. This result could be due to a lack of statistical power, but we cannot exclude that the S127R mutation might modulate LDLR expression independently of SREBP2 signaling.

hiPSCs have already been used as a model for studying ADH linked to LDLR mutations (Cayo et al., 2012; Idriss et al., 2015; Rashid et al., 2010). Here, we showed for the first time that HLCs differentiated from urine-derived hiPSCs are suitable to model ADH related to a PCSK9 GOF mutation. The main advantages of urine collections are their ease of use, the very low occurrence of contamination (about 6%) and, in cases in which the first sample did not successfully yield somatic cells or efficient reprogramming, it is easier to obtain new urine samples compared with obtaining other sources of somatic cells such as skin biopsies or blood samples. More importantly, because it is a non-invasive method, it provides a better acceptability by the patient compared to skin biopsies, and seems to be suitable for accessing cells from pediatric patients with minimal stress.

### Conclusions

We demonstrated for the first time that UhiPSC-derived hepatocytes are a valuable and reproducible human *in vitro* cellular model to study PCSK9 biology. This model will certainly help to further decipher the function of PCSK9 mutations, to identify or validate new PCSK9 partners and to assess the role of PCSK9 in lipoprotein(a) metabolism (Romagnuolo et al., 2015). Ultimately, such UhiPSC-derived hepatocytes are a promising pharmacological model to test new PCSK9 inhibitors or modulators such as the novel Sirt1 activator SRT3025, which has been recently shown to reduce PCSK9-mediated LDLR degradation (Miranda et al., 2015).

## MATERIALS AND METHODS

### Ethics statement

The study was conducted in compliance with current Good Clinical Practice standards and in accordance with the principles set forth under the Declaration of Helsinki (1989). Each subject entering the study agreed to and signed an institutional-review-board-approved statement of informed consent for the collection of urine samples (authorization number from the French Ministry of Health: DC-2011-1399).

### Ucell collection and culture

Urine samples were collected in a 250 ml bottle previously conditioned with 10% of RE/MC medium (see below) for storage (up to 24 h at 4°C) and transported as previously described (Lang et al., 2013). RE/MC (1:1) medium was prepared as described by Zhou et al. (2012) by mixing RE medium (Renal epithelial cell growth medium SingleQuot kit supplement and growth factors; Lonza) with MC (mesenchymal cell) medium prepared separately. MC medium is composed of DMEM/high glucose medium (Hyclone) supplemented with 10% (vol/vol) FBS (Hyclone), 1% (vol/vol) GlutaMAX (Life Technologies), 1% (vol/vol) NEAA (Life Technologies), 100 U/ml penicillin (Life Technologies), 100 μg/ml streptomycin (Life Technologies), 5 ng/ml bFGF2 (Miltenyi), 5 ng/ml PDGF-AB **(**Cell Guidance Systems) and 5 ng/ml EGF (Peprotech). Ucells were isolated from urine samples and cultured according to the procedure described in Zhou et al. (2012) with slight modifications. Briefly, urine samples were centrifuged 5 min at 1200 ***g*** and the pellet was washed with pre-warmed DPBS (Gibco) containing 100 U/ml penicillin and 100 µg/ml streptomycin (Gibco). Pellets were resuspended in 2 ml RE/MC proliferation medium and cultured on 0.1% gelatin-coated six-well plates. Cells were incubated at 37°C in normoxia (20% O_2_, 5% CO_2_) for 4-5 days without any change of medium nor moving. Ucells were further passaged using TrypLE Express (Gibco) and expanded in RE/MC medium with daily change of half of the media. Upon amplification, Ucells were characterized and frozen (Biobanker, Zenoaq). For Ucell characterization, expression of mesenchymal stem cells surface markers (see Table S1) was measured using flow cytometry and their capacity to differentiate into osteocytic and chondrocytic tissue was evaluated as previously described (Bharadwaj et al., 2013).

### Ucell reprogramming

Ucells were reprogrammed into UhiPSCs as follow. Briefly, 3×10^5^ to 5×10^5^ of fresh or frozen Ucells were nucleofected using the basic epithelial cells Nucleofector Kit (Lonza) with episomal vectors coding for OCT4, SOX2, KLF4, MYC, LIN28, NANOG and SV40LT (Addgene Cat# 20922, 20923, 20924, 20925 and 20927), and a non-episomal vector coding for miR302/367 (System Biosciences Cat# TDH101PA-GP). Nucleofected Ucells were cultured at 37°C under hypoxia conditions (4% O_2_, 5% CO_2_) for 7 days with RE/MC medium then 14-21 days with TeSR-E7 medium (Stem Cell Technologies**)**. UhiPSC clones were manually picked and transferred onto mitomycin-treated MEFs for further culture and amplification.

### UhiPSC culture

UhiPSCs were cultured on MEFs in hiPS medium composed of DMEM-F12 medium (Life Technologies) supplemented with 20% Knockout Serum Replacer (Life Technologies), 0.5% L-Glutamine (Life Technologies) supplemented with 0.14% 2-Mercaptoethanol (Sigma), 1% NEAA and 5 ng/ml of fibroblast growth factor 2 (FGF2; Miltenyi) under hypoxic condition (4% O_2_, 5% CO_2_) and manually passed once a week. For feeder-free culture conditions, UhiPSC colonies were manually dissected from MEFs and plated onto plates coated with Matrigel (Corning; 0.05 mg/ml) in mTSeR (Stem Cell Technologies) or StemMACS iPS-Brew medium (Miltenyi). Passages were performed using the Gentle Cell Dissociation Buffer (Stem Cell Technologies).

### Teratoma formation

Undifferentiated UhiPSCs cultured on MEFs were mechanically dissociated with EZ Passage tools (Life Technologies), resuspended in Matrigel (2 mg/ml) and injected subcutaneously into NOD Scid gamma (NSG) mice. Tumor samples were collected at 8-10 weeks, fixed in 4% paraformaldehyde, embedded in paraffin, sectioned, and stained with hematoxylin and eosin for analysis.

### Karyotyping

UhiPSCs were grown on Matrigel until reaching a 70% confluence and treated with 0.13 µg/ml Colcemid (Life Technologies). UhiPSCs were then removed, washed and subjected to a hypotonic shock using 0.075 M KCl, before fixation with a 3:1 mixture of methanol/acid acetic. Pellets were washed and sent for analysis at Cell Guidance Systems (http://www.cellgs.com/Shop/Services/Karyotyping.html).

### UhiPSC hepatic differentiation

UhiPSCs from passage 25-40 were differentiated into HLCs (named hepatocyte in figures) as previously described (DeLaForest et al., 2011; Si-Tayeb et al., 2010). Briefly, UhiPSCs were plated in tissue-culture plates previously coated with Matrigel at 0.05 mg/ml for 1 h, and cultured in mTSeR or StemMACS iPS-Brew. Once cells reached 70-80% confluence, differentiation was performed with RPMI 1640 (Life Technologies) and B27 (Life Technologies) containing Activin A (AA; Miltenyi), FGF2, bone morphogenetic protein 4 (BMP4; Miltenyi) and hepatocyte growth factors (HGFs; Miltenyi) with the following sequence: AA/BMP4/FGF2 (2 days); AA (3 days), BMP4/FGF2 (5 days) and HGF (5 days). Cells were then incubated with Hepatocyte Culture Medium (Lonza) supplemented with oncostatin M (Miltenyi) for 5 days. The hepatic differentiation efficiency for each UhiPSC culture was determined upon counting of albumin-positive cells compared to total cells from multiple fields.

### Immunostainings

Cultured cells were fixed with 4% paraformaldehyde for 30 min at room temperature (RT), permeabilized with 0.5% Triton X-100 in PBS for 15 min and blocked with 3% BSA in PBS for 30 min. Cells were incubated overnight at 4°C with primary antibodies diluted in 1% BSA in PBS, washed three times with PBS/BAS 1% and incubated with suitable secondary antibodies for 1 h at RT together with DAPI. Antibody references and dilutions are listed in Table S1. Immunostainings were examined using an inverted epifluorescent microscope (Zeiss Axiovert 200M).

### Flow cytometry

For flow-cytometry analysis, 10^5^ cells in suspension for each condition were washed three times with a FACS Buffer (PBS/BSA 0.1%) then incubated with PE-labeled antibodies (see Table S1) for 30 min at 4°C in the dark and further rinsed three times with FACS buffer. Data acquisition was performed using FACSDiva software with an LSR II instrument (Becton Dickinson) or the Accury C6 (Becton Dickinson).

### Genomic sequencing

Genomic DNA was isolated using the NucleoSpin Tissue Purification Kit (Macherey Nagel). The DNA fragment of exon2 *PCSK9* for S127R mutation sequencing was amplified by PCR using 100 ng of genomic DNA and specific forward (5′-TGGTCCGCATTTGGTAACTT-3′) and reverse (5′-CCCCTTCTGATTTTCAGCAAT-3′) primers. PCR products were then sequenced using the AB3730 sequencer (Applied Biosystems).

### Gene expression analysis

RNA samples were isolated by using the RNeasy Mini Kit (Qiagen). Reverse transcription of 1 μg of RNA into cDNA was conducted using the High-Capacity cDNA Reverse-Transcription Kit (Applied Biosystems). Conditions were as follows: 10 min at 25°C, and then 2 h at 37°C. Quantitative polymerase chain reaction (qPCR) studies were conducted in triplicate by using the Brilliant III Ultra-Fast Master Mix with high ROX (Agilent). Primers are listed in Table S2. Each qPCR included 2 s at 50°C, 10 s at 95°C followed by 40 cycles of 15 s at 95°C, and 60 s at 60°C. Cycle threshold was calculated by using default settings for the real-time sequence detection software (Applied Biosystems).

### Episomal vector detection

RT-PCR reactions were conducted in triplicate by using the Mesagreen 2× PCR Master Mix for SYBR (Eurogentec). The following primers were used: forward: 5′-GGCTCTCCCATGCATTCAAA-3′, reverse: 5′-GGCCCTCACATTGCCAAA-3′, with DNA concentration at 5 ng/well. Conditions were: 2 s at 90°C, followed by 40 cycles of 10 s at 95°C and 30 s at 60°C, and finished by 60 s at 60°C. Cycle threshold was calculated by using default settings for the real-time sequence detection software (Applied Biosystems).

### Gene arrays

RNA samples were prepared and hybridized on Agilent Human Gene Expression 8×60 K microarrays (Agilent Technologies, part number: G4851A). Normalization procedures were performed using R statistical software (http://www.r-project.org). The raw signals of all probes for all the arrays were normalized against a virtual median array (median raw intensity per row) using a local weighted scattered plot smoother analysis (LOWESS). The data were filtered to remove probes with low intensity values. This filtering is performed by sample category in order to keep the signature of categories with a small sample size. A hierarchical clustering was computed on median-gene-centered and log-transformed data using average linkage and uncentered correlation distances with the Cluster program. Raw and normalized data have been deposited in the Gene Expression Omnibus (GEO) database (accession number GSE75545).

### ELISA assay

PCSK9 levels in conditioned medium were assayed in duplicates using a commercially available quantitative sandwich ELISA assay following the manufacturer’s instructions (Circulex CY-8079, CycLex Co.). Data were presented as PCSK9 secretion during 24 h per well (of a six-well plate) of differentiated cells.

### LDL uptake assay

Hepatocytes were exposed for 3 h to 5 µg/ml dil-LDL (Life Technologies) or 5 µg/ml of BODIPY-FL-LDL (Life Technologies) in HCM medium. For uptake measurement, hepatocytes were washed five times with PBS then dissociated for 40 min in Gentle Cell Dissociation Reagent. Cells were recovered in PBS and analyzed by flow cytometry using the FACS ARIA III (Becton Dickinson).

### Statistical analysis

Data are expressed as mean±s.e.m. Significant differences between mean values were determined with the Mann–Whitney *U*-test for comparison of two groups or paired Student’s *t*-test if appropriate. For the cluster approach, genes belonging to the same biological function or cell type are known to exhibit correlated expression. We use hierarchical clustering to detect groups of correlated genes supported by a statistical method (limma) to detect differential expression among biological conditions.
